# PPAR*γ* Antagonizes Hypoxia-Induced Activation of Hepatic Stellate Cell through Cross Mediating PI3K/AKT and cGMP/PKG Signaling

**DOI:** 10.1155/2018/6970407

**Published:** 2018-03-01

**Authors:** Qinghui Zhang, Shihao Xiang, Qingqian Liu, Tao Gu, Yongliang Yao, Xiaojie Lu

**Affiliations:** ^1^Department of Clinical Laboratory, Kunshan First People's Hospital, Affiliated to Jiangsu University, Kunshan, Jiangsu Province 215300, China; ^2^Department of Gastroenterology, Shanghai Tongren Hospital, Shanghai Jiaotong University School of Medicine, Shanghai 200336, China; ^3^Department of Liver Surgery, The First Affiliated Hospital of Nanjing Medical University, Nanjing, China

## Abstract

**Background and Aims:**

Accumulating evidence reveals that PPAR*γ* plays a unique role in the regulation of hepatic fibrosis and hepatic stellate cells (HSCs) activation. This study was aimed at investigating the role of PPAR*γ* in hypoxia-induced hepatic fibrogenesis and its possible mechanism.

**Methods:**

Rats used for CCl4-induced hepatic fibrosis model were exposed to hypoxia for 8 hours each day. Rats exposed to hypoxia were treated with or without the PPAR*γ* agonist rosiglitazone. Liver sections were stained with HE and Sirius red staining 8 weeks later. HSCs were exposed to hypoxic environment in the presence or absence of rosiglitazone, and expression of PPAR*γ* and two fibrosis markers, *α*-SMA and desmin, were measured using western blot and immunofluorescence staining. Next, levels of PPAR*γ*, *α*-SMA, and desmin as well as PKG and cGMP activity were detected using PI3K/AKT and a cGMP activator or inhibitor.

**Results:**

Hypoxia promoted the induction and progress of hepatic fibrosis and HSCs activation. Meanwhile, rosiglitazone significantly antagonized the effects induced by hypoxia. Signaling by sGC/cGMP/PKG promoted the inhibitory effect of PPAR*γ* on hypoxia-induced activation of HSCs. Moreover, PI3K/AKT signaling or PDE5 blocked the above response of PPAR*γ*.

**Conclusion:**

sGC/cGMP/PKG and PI3K/AKT signals act on PPAR*γ* synergistically to attenuate hypoxia-induced HSC activation.

## 1. Introduction

Fibrosis is a common response to hepatic damage, which is characterized by producing and depositing extracellular matrix (ECM) [1]. Excessive fibrosis characterizes a series of liver diseases, such as chronic hepatitis, alcoholism-induced liver damage, and hepatic autoimmune disorders [2]. During this pathological process, hepatic stellate cells (HSCs) are the main executor of fibrogenesis. HSC activation increases the expression and secretion of collagen and other ECM components. It also stimulates hepatic microenvironment cells, such as macrophages, endothelial cells, and inflammatory cells, resulting in the promotion of fibrogenesis in an autocrine or paracrine manner [3, 4]. Therefore, understanding the molecular mechanism based on HSC activation is essential for diagnosis and treatment of hepatic fibrosis.

Peroxisome proliferator-activated receptor (PPAR)*γ* is a fundamental nuclear receptor that regulates lipid metabolism, insulin sensitivity, and fat deposition. It plays an extremely important role in liver physiological metabolism [5]. However, increasing researches have implicated that PPAR*γ* is a key mediator in HSC activation and phenotypic alteration, thus maintaining HSCs in a quiescent phase [6, 7]. Recently, oxidative stress is considered to be involved in HSC activation and hepatic fibrogenesis [8]. A large amount of publications has also reported that HSCs exposed to hypoxia could be activated through HIF1*α* and its downstream target genes or signaling pathways [9–11]. Based on those evidences, we hypothesized that the effect of PPAR*γ* on HSCs is the mechanisms underlying the role of hypoxia in liver fibrogenesis. In fact, PPAR*γ* has been found to be regulated by hypoxia in several diseases. Wang et al. reported that hypoxia decreased UCP2 via HIF-1-mediated suppression of PPAR*γ*, leading to chemoresistance of non-small cell lung cancer [12]. Jiang et al. found that hypoxia inhibited PKG-PPAR*γ* axis in rat distal pulmonary arterial smooth muscle cells (PASMCs) and distal pulmonary arteries [13]. However, it is not clear whether these cellular events are associated with the pathogenesis of hepatic fibrosis and its key functionary mechanisms. We therefore performed* in vivo* and* in vitro* experiments to test the above hypothesis.

## 2. Materials and Methods

### 2.1. Animals and Experimental Protocol

This study was performed on 35 male SD rats with weight of 200–250 g from Shanghai SLAC Laboratory Animal Co. Ltd. (Shanghai, China), housed in regular cages, situated in an animal room at 22 ± 2°C and maintained on a 14-hour light/10-hour dark cycle. The rats were randomly divided into 4 groups as follows: Group I (*n* = 5), serving as controls, were maintained in a standard normoxic chamber (FiO_2_ 0.21). Group II (*n* = 10) rats were injected with 40% CCl4 (the mixture of CCl4 and olive oil) in a standard normoxic chamber (FiO_2_ 0.21). Group III (*n* = 10) rats were injected with 40% of CCl4 mixture in normobaric hypoxic chamber (FiO_2_ 0.07) with hypoxia exposures 8 hours each day. Group IV (*n* = 10) were modeled in the same manner as Group III (40% CCl4 mixture injection plus hypoxia exposures) but treated with PPAR*γ* agonist rosiglitazone (RSG). RSG was added to the liquid diet at a daily intake of 10 mg/kg body wt for feeding.

All rats were sacrificed after 8 weeks, and blood was collected from the abdominal aorta. Serum was separated and stored at −80°C for measurement of hyaluronic acid (HA), laminin (LN), N-terminal peptide of type III procollagen (PIIINP), and collagen type IV (CIV). Then, liver was immediately removed. One part of the liver was used for the extraction of protein for western blotting. The other part of liver was fixed in 4% paraformaldehyde, embedded in paraffin, and cut into sections (5 *μ*m thick) for HE and Sirius red staining. Animal care and procedures were approved by Medical Ethics Committee of Jiangsu University, China.

### 2.2. Reagents

8-Br-cGMP, Rp-8-Br-PET-cGMPS, LY294002, rosiglitazone, and zaprinast were purchased from Sigma-Aldrich. 740 Y-P was supplied by R&D systems. LY294002, rosiglitazone, and zaprinast were dissolved in dimethyl sulfoxide (final concentration 0.2%). The other drugs were prepared using distilled water.

### 2.3. Serum Parameters Examination

Serum levels of HA, LN, PIIINP, and CIV were detected by using ELISA Kits (Elabscience, Wuhan, China) according to the manufacturer's instruction.

### 2.4. HE and Sirius Red Staining

Tissue slices were fixed in 4% paraformaldehyde, embedded with paraffin, and sectioned using standard techniques. Sections were subjected to HE and Sirius red staining. Each sample was assessed independently and scored by two pathologists for a blind evaluation, according to the modified Scheuer fibrosis score system [14, 15]. The fibrosis stage score was categorized into five stages (0–4): 0: none, 1: zone 3 perisinusoidal fibrosis; 2: zone 3 perisinusoidal fibrosis plus portal fibrosis; 3: perisinusoidal fibrosis and portal fibrosis, plus bridging fibrosis; and 4: cirrhosis.

### 2.5. Cell Culture

Rat HSC-T6 cell line was a gift from Dr. Ke AiWu (Liver Cancer Institute, Zhongshan Hospital, Fudan University, China). Cells were maintained in Dulbecco's modified Eagle's medium (Hyclone, USA) containing 10% fetal bovine serum (Hyclone, USA), penicillin (100 IU/ml), and streptomycin (100 IU/ml) (Amresco, USA). Cells were cultured at 37°C in a humidified atmosphere of 5% CO2.

### 2.6. Measurement of Total Cellular cGMP

The supernatant of HSC-T6 cells were collected and stored at −80°C. Total levels of cGMP were measured using a competitive ELISA assay (Cayman Chemical, USA) according to the manufacturer's recommendations.

### 2.7. Measurement of PKG Activity

The activity of PKG including PKG-I and PKG-II in each sample was measured according to the protocol of the ELISA kit (CycLex, Japan), in which the phosphorylated antibody can identify the phosphorylation of threonine (Thr 68/119) residues on substrate of PKG.

### 2.8. Immunofluorescence Staining

Immunofluorescence staining assays were performed as we described previously [15]. Cells were incubated overnight with the primary antibody against *α*-SMA (ab5694) and desmin (ab15200) (1 : 50 dilution; Abcam, USA) at 4°C. The cells were washed three times with PBS (5 min each) and then incubated in the dark for 1 h at room temperature with Alexa Fluor 488 and 550 conjugated goat anti-rat secondary antibody (1 : 200 dilution; ab150157 and ab150083, Abcam, USA). Nuclei of cells were stained with 4′,6-diamidino-2-phenylindole (DAPI) (Sigma-Aldrich, USA). Images were obtained using a Zeiss LSM 510 META Confocal microscope using 20x/0.5 w and 40x/1.2 w objectives.

### 2.9. Western Blot Analysis

Western blot analysis was performed as described in a previous report from ours [16]. Primary antibodies used in this study are as follows: PPAR*γ* (ab209350) (1 : 1,500), *α*-SMA (ab5694), desmin (ab15200), AKT (ab8805), Phospho-AKT (ab81283), PI3K p110*β* (ab151549), PDE5 (ab14672) (1 : 1,000; ABcam, USA), and PI3K p110*α* (#4249) (1 : 1,200; Cell Signaling Technology, USA). The primary antibody of GAPDH was diluted at 1 : 1,000–1,500 (Santa Cruz, USA). The secondary HRP-conjugated antibodies were diluted at 1 : 2,500. The blots were detected using the ECL system (Beyotime Biotechnology, China). A LI-COR Odyssey scanner (LICOR) was used to analyze the intensity of bands on the blots.

### 2.10. Statistical Analysis

All statistical analyses were performed using SPSS 17.0 software. Data are expressed as mean ± SE, and mean values were compared using the Student's* t*-test and ANOVA. Data were as mean ± SE. Western blot, ELISA, PKG activity, and cGMP analysis were examined using one-way ANOVA. The *χ*^2^ and Fisher's exact test were used to analyze the differences of fibrosis grades in each group. Values of *P* < 0.05 were considered statistically significant.

## 3. Results

### 3.1. Low Expression of PPAR*γ* Is Associated with Hypoxia-Induced Hepatic Fibrosis

**Figure 1:**
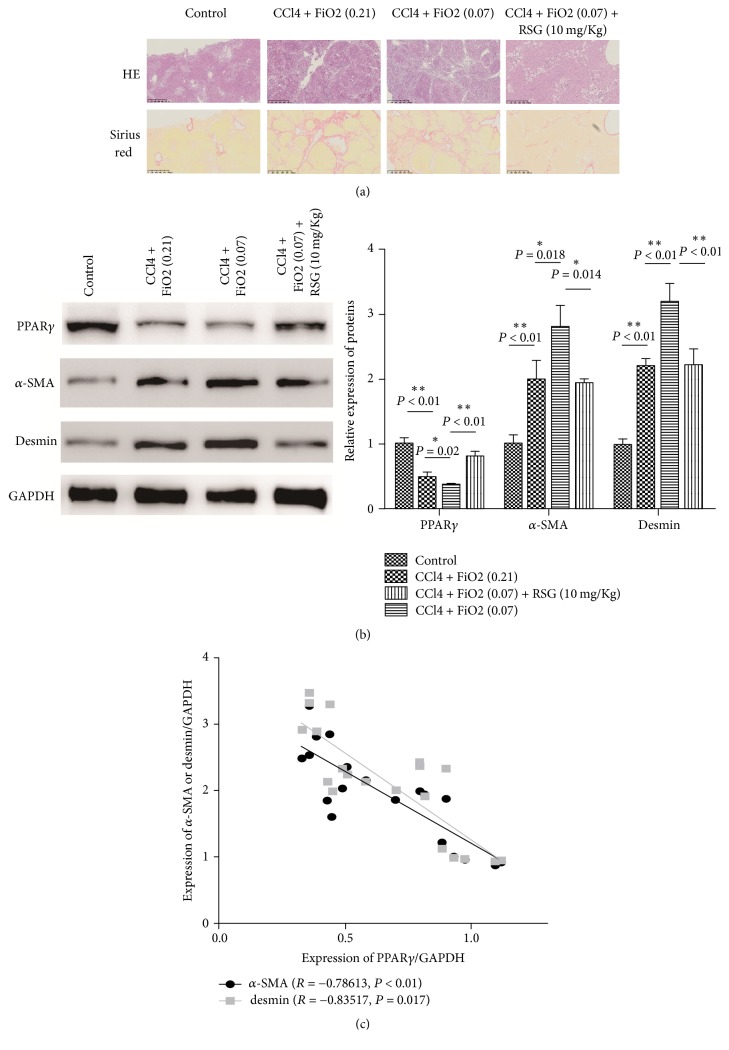
Effects of PPAR*γ* on hypoxia-induced hepatic fibrosis. (a) HE and Sirius red staining for liver tissues (magnification, ×200). (b) PPAR*γ*, *α*-SMA, and desmin expression of liver tissues in experimental groups were detected by western blotting analysis. (c) The correlation between the expression levels of PPAR*γ* and *α*-SMA or desmin. Bars stand for mean ± SD (same for all figures). For each assay, *n* = 3. ^*∗*^*P* < 0.05, ^*∗∗*^*P* < 0.01.

Consistent with previous studies, we found that hypoxia can promote the progress of hepatic fibrosis (Tables 1 and 2 and Figure 1). Meanwhile, the PPAR*γ* agonist rosiglitazone (RSG) significantly antagonized hypoxia-induced development of hepatic fibrosis. Hypoxia resulted in a trend towards increasing serum markers (HA, LN, PIIINP, and C IV), but also elevated fibrosis histological grade. However, the activation of PPAR*γ* ameliorated this effect (Tables 1 and 2 and Figure 1). To examine the possible correlation of PPAR*γ* with hepatic fibrosis, the correlation between the expression levels of PPAR*γ* and other two markers of fibrosis, *α*-SMA and desmin, was measured. As shown in Figures 1(b)-1(c), PPAR*γ* was negatively correlated with *α*-SMA and desmin expression (*r* = −0.78613,* P* < 0.001, and* r* = −0.83517,* P* = 0.017, resp.). These results suggest that PPAR*γ* is associated with hypoxia-induced hepatic fibrosis.

### 3.2. PPAR*γ* Antagonizes HSCs Activation Caused by Hypoxic Stress

**Figure 2:**
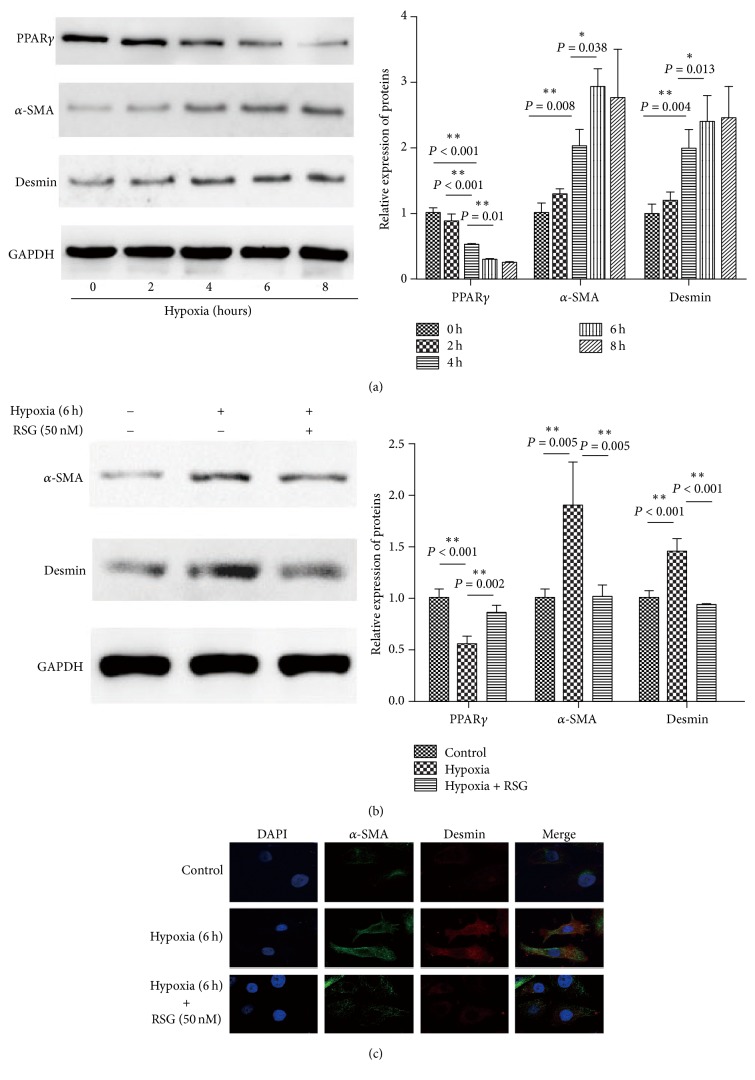
PPAR*γ* inhibits hypoxic-induced HSCs activation. (a) HSCs were exposed to hypoxia for the designated time. Protein expressions of PPAR*γ*, *α*-SMA, and desmin were tested by western blotting. HSCs were exposed to hypoxia for 6 hours in the presence or absence of PPAR*γ* agonist, rosiglitazone (RSG, 50 nM). (b) PPAR*γ*, *α*-SMA, and desmin expression of HSCs in experimental groups were analyzed by western blotting. (c) *α*-SMA and desmin expression in HSCs in experimental groups were detected by immunofluorescence using Confocal microscopy. For each assay, *n* = 3. ^*∗*^*P* < 0.05, ^*∗∗*^*P* < 0.01.

It is well known that HSCs are important effectors inducing hepatic fibrosis. Previously, we demonstrated that hypoxia promoted hepatic fibrogenesis through the regulation of PPAR*γ in vitro*. To clarify if HSC is a modulator of this process, HSC was exposed to hypoxic stress with oxygen concentration decreased from 21% to 7% at different time. In comparison to control groups, PPAR*γ* protein level was significantly decreased in a time-dependent manner, accompanied by an obvious increased expression in two markers of fibrosis, *α*-SMA and desmin (Figure 2(a)). At the same time, based on our observation, the trend reached plateau after 6 hours of hypoxia exposure. These results demonstrated that the early phase of hypoxic stress might cause HSCs activation.

Then, the expression of *α*-SMA and desmin were analyzed in HSCs exposed to hypoxia in the presence or absence of the PPAR*γ* agonist, rosiglitazone (RSG, 50 nM), by western blotting and immunofluorescence. As expected, hypoxia exposure to HSCs alone led to significant PPAR*γ* downregulation and *α*-SMA and desmin rise, whereas cotreatment with hypoxia and RSG reversed these effects (Figure 2(b)), indicating that PPAR*γ* antagonizes HSCs activation caused by hypoxic stress.

### 3.3. PI3K/AKT Signals Involved in Hypoxia-Induced PPAR*γ* Low Expression

**Figure 3:**
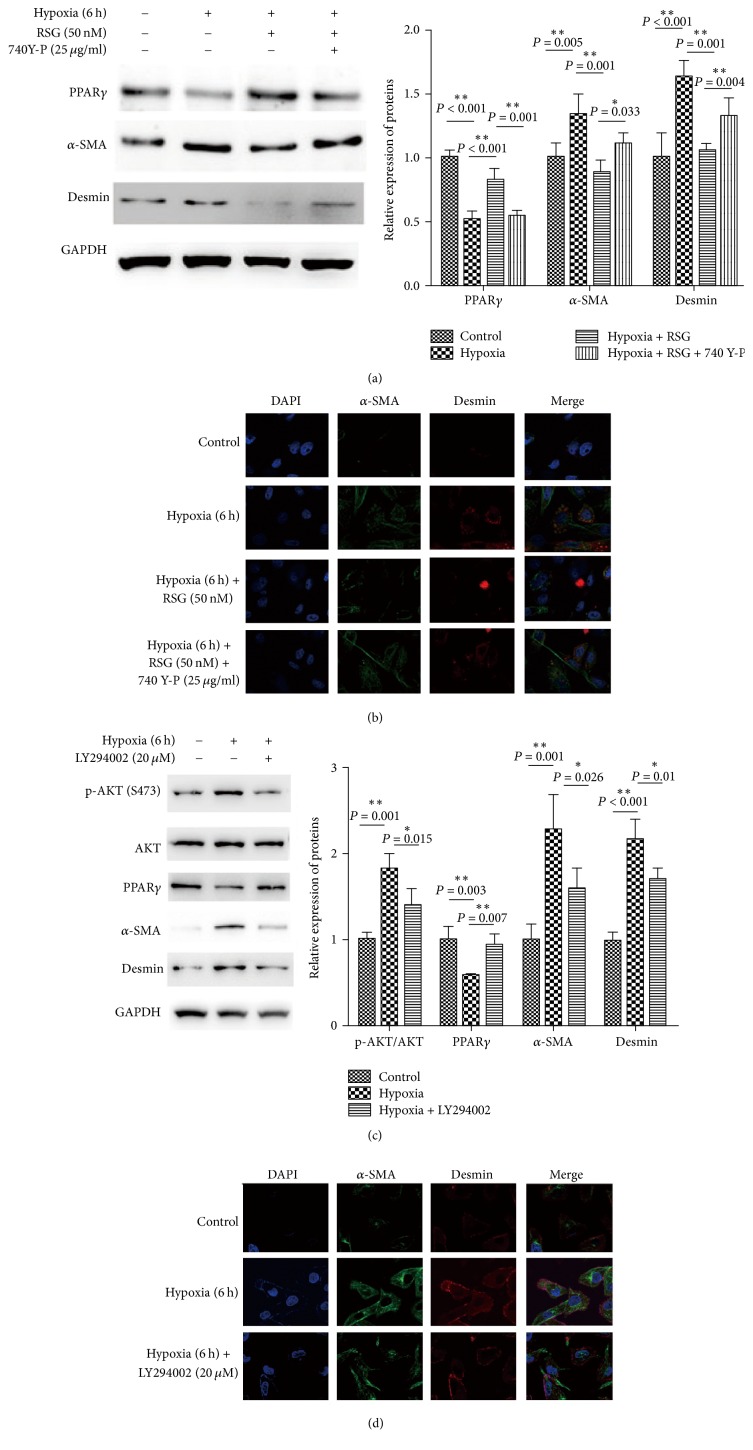
PI3K/AKT signaling blocks the inhibitory effect of PPAR*γ* on hypoxic-induced HSCs activation. Activation of PI3K with 740 Y-P (25 *μ*g/ml) of HSCs exposed to hypoxia in the presence or absence of RSG (50 nM). (a) PPAR*γ*, *α*-SMA, and desmin expression of HSCs in experimental groups were analyzed by western blotting. (b) *α*-SMA and desmin expression of HSCs in experimental groups were detected by immunofluorescence using Confocal microscopy. Inhibition of PI3K with LY294002 (20 *μ*M) in HSCs exposed to hypoxia. (c) Phosphorylated AKT, total AKT, PPAR*γ*, *α*-SMA, and desmin expression of HSCs in experimental groups were measured by western blotting. (d) *α*-SMA and desmin expression of HSCs in experimental groups were detected by immunofluorescence using Confocal microscopy. For each assay, *n* = 3. ^*∗*^*P* < 0.05, ^*∗∗*^*P* < 0.01.

The PI3K/AKT signaling pathway is an essential mechanism by which cells regulate oxidative stress effect and induces HSC activation and proliferation dependent on HIF-1*α* in response to hypoxia. Here, we analyzed whether PI3K/AKT signaling is associated with PPAR*γ* inhibition of hypoxia-induced HSCs activation. Results showed that hypoxia-induced HSCs activation when AKT phosphorylation was significantly enhanced (Figure 3(c)). Inhibition of PI3K with LY294002 (20 *μ*M) distinctly opposed the above-mentioned activation. Simultaneously, PPAR*γ* expression was also recovered (Figures 3(c) and 3(d)). Similarly, activation of PI3K with 740 Y-P (25 *μ*g/ml) of HSCs exposed to hypoxia in the presence of RSG (50 nM) significantly reduced PPAR*γ* expression, along with increased level of *α*-SMA and desmin (Figures 3(a) and 3(b)). These results imply that PI3K/AKT signaling blocked the inhibitory effect of PPAR*γ* on hypoxia-induced activation of HSCs.

### 3.4. The Cross Talk of sGC/cGMP/PKG and PI3K/AKT Signaling Adjusts PPAR*γ* Attenuating Hypoxia-Induced HSC Activation

**Figure 4:**
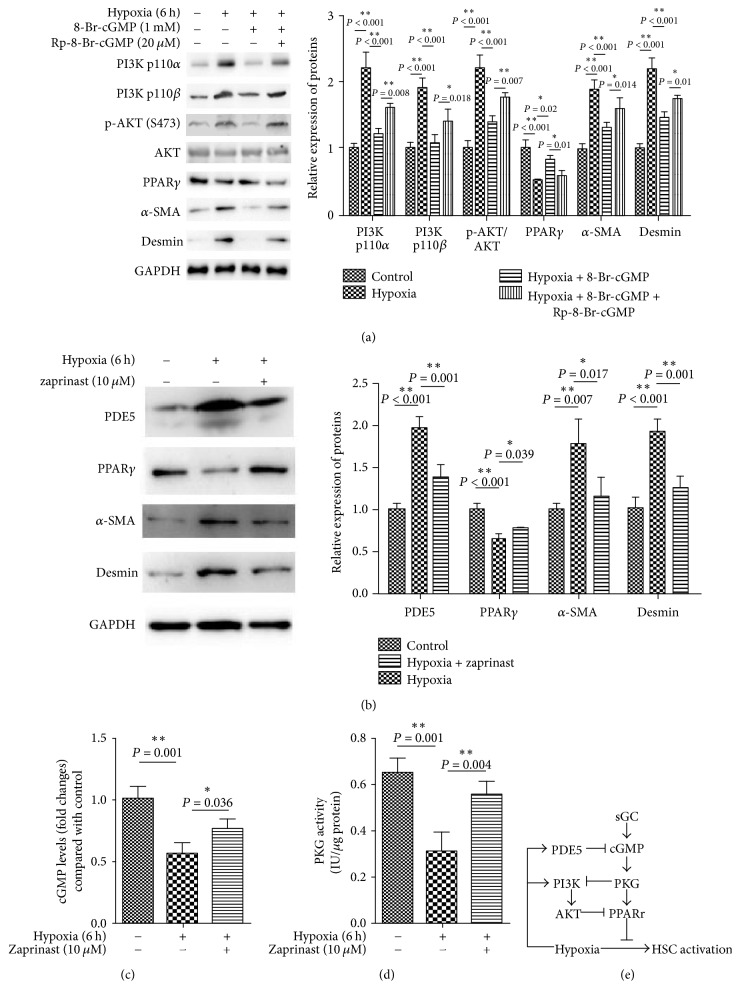
sGC/cGMP/PKG and PI3K/AKT signals synergistically act on PPAR*γ* to inhibit hypoxia-induced HSC activation. (a) HSCs were exposed to hypoxia in the presence of cGMP analog, 8-Br-cGMP (1 mM), or agonist, Rp-8-Br-cGMPs (20 *μ*M). PI3K p100 *α*, p100*β*, phosphorylated AKT, total AKT, PPAR*γ*, *α*-SMA, and desmin expression of HSCs in experimental groups were detected by western blotting. HSCs were exposed to hypoxia in the presence of PDE5 agonist, zaprinast (10 *μ*M). (b) PDE5, PPAR*γ*, *α*-SMA, and desmin expression of HSCs in experimental groups were measured by western blotting. (c)-(d) cGMP and PKG activity were tested, respectively. (e) Possible signal pathway involved in this study. Hypoxia induces HSCs activation. However, PPAR*γ* inhibits this effect, which is triggered by sGC/cGMP/PKG signaling. Hypoxia promotes PI3K/AKT signaling and PDE5, which inhibit cGMP and PPAR*γ*, respectively. Finally, sGC/cGMP/PKG and PI3K/AKT signals synergistically act on PPAR*γ* to inhibit hypoxia-induced HSC activation. For each assay, *n* = 3. ^*∗*^*P* < 0.05, ^*∗∗*^*P* < 0.01.

According to previous studies, sGC/cGMP/PKG contributes to the treatment of cirrhosis. In addition, this signal also modulates various cellular events induced by hypoxic stress. To investigate the potential role of sGC/cGMP/PKG signaling in HSC hypoxia responses modulated by PPAR and PI3K/AKT signaling, HSCs were incubated for 90 min under hypoxia with or without 8-Br-cGMP (1 mM) which was widely used as sGC/cGMP/PKG agonist and in the presence or absence of Rp-8-Br-cGMP (20 *μ*M), a sGC/cGMP/PKG antagonist. We found that cotreated 8-Br-cGMP with hypoxia showed a significant decrease in protein levels of PI3K p110*α*, PI3K p110*β*, and phosphorylated AKT in comparison with the hypoxia group. In consequence of this decline, PPAR*γ* expression increased. Moreover, just as we speculated, *α*-SMA and desmin level also reduced. The effect induced by 8-Br-cGMP was blocked by the inclusion of Rp-8-Br-cGMP (Figure 4(a)). These results indicated that sGC/cGMP/PKG directly inhibited PI3K/AKT signaling and on the other hand increased PPAR*γ*, eventually affecting the hypoxia-induced HSCs activation.

To further confirm the above findings, we used zaprinast, a specific inhibitor of phosphodiesterase type 5 (PDE5), to inhibit cGMP hydrolysis and to observe the subsequent effects on PPAR*γ* and PI3K/AKT signaling on hypoxia-induced HSC activation. Results showed that the hypoxic environment significantly improved the cGMP and PKG activity in HSCs (Figures 4(c)-4(d)). PDE5 inhibition restored cGMP and PKG activity (Figures 4(c)-4(d)), followed by increased PPAR*γ* expression and repressed HSCs activation (decreased expression of *α*-SMA and desmin) (Figure 4(b)). These data suggested that sGC/cGMP/PKG and PI3K/AKT signals synergistically act on PPAR*γ*, and the latter inhibited hypoxia-induced HSC activation.

## 4. Discussion

PPAR*γ*, as a transcription factor, controls gene transcription and cell differentiation both* in vitro* and* in vivo* and plays a key role in inhibiting HSC activation and maintaining the morphological and biochemical reversal of activated HSC to quiescent cells [17–19]. Current evidences have shown that hypoxia is a common stimulus to the development of hepatic fibrosis and HSCs activation [20, 21]. In fact, hypoxia is capable of connecting a variety of signals. It not only affects the activation of HSCs but also acts on a variety of other cells, such as sinusoidal cells, hepatocytes, adipocytes, and liver-resident macrophages (Kuppfer cells) [1, 22–24]. Secondly, hypoxia-induced structural changes in hepatic fibrosis aggravate the hypoxic environment, thus, in turn, accelerating its pathologic progression of hepatic fibrosis.

However, little is known regarding the effect of PPAR*γ* on HSC activation and hepatic fibrosis under hypoxia stress. To elucidate the interrelationship and molecular mechanism between HSCs activation and PPAR*γ* in hypoxic environment, we designed experiments both* in vitro* and* in vivo*. For this purpose, by exposing CCl4 modeling rats to hypoxic environment, we validated the hypothesis that hypoxic stress promoted hepatic fibrosis. Notably, expression of PPAR*γ* was inhibited by hypoxia. Moreover, expression of two markers of fibrosis, *α*-SMA and desmin, significantly increased. To further explore the effects of PPAR*γ* in hypoxia, RSG, a PPAR*γ* agonist, was added to hypoxia exposed groups. As expected, hepatic fibrosis induced by hypoxia was ameliorated with increasing PPAR*γ* level. These results were also confirmed by HSC experiments* in vivo*. Our findings confirmed that PPAR*γ* antagonizes hepatic fibrosis caused by hypoxia stress through inhibiting HSCs activation.

However, the underlying mechanism that PPAR*γ* mediated hypoxia-induced HSC activation must be further explored. Recent studies displayed that AKT signaling and PPAR*γ* have a mutual inhibitory effect [25–27], but this theory is still controversial. Kilter et al. suggested that PPAR*γ* facilitates AKT phosphorylation in cardiomyocytes during hypoxia/reoxygenation [28]. However, another independent study found that PPAR*γ* inhibits endothelial cell migration by inhibiting PI3K/AKT signaling [29]. Our present study showed that PI3K/AKT signaling blocked the inhibitory effect of PPAR*γ* on hypoxia-induced HSCs activation. Next, cGMP analogs and antagonists were used, and results showed that sGC/cGMP/PKG directly inhibited PI3K/AKT signaling and increased PPAR*γ* expression, eventually affecting the hypoxia-induced HSCs activation. In contrast, inhibition of PDE5 and resisting cGMP hydrolysis restore cGMP and PKG activity, followed by increased PPAR*γ* expression and repressed HSCs activation.

Given the synergistic effect of hypoxia and insulin resistance in liver fibrosis, previous study showed that hypoxia leads to a decrease in PPAR*γ* expression, followed by inhibition of IR transcription, resulting in the blockade of IGF-1 and subsequent signals. When TZD restores PPAR*γ*, IR activation occurs directly and indirectly, initiating IGF-1/PI3K signaling [30]. However, we found that the hypoxic environment induced the PI3K signals, leading to the decrease of PPAR*γ* expression, indicating that there is a complex negative feedback mechanism in this process.

Based on these data, we speculated that sGC/cGMP function on PKG activation to activate PPAR*γ*, which attenuates hypoxia-induced HSCs activation and hepatic fibrogenesis. On the other side, hypoxia directly and indirectly inhibits the expression of PPAR, by inducing PI3K/AKT signaling or activating PDE5 to cGMP hydrolysis, which is ultimately beneficial to hypoxia-induced HSCs activation. Our findings may provide new insights into the mechanisms leading to fibrosis in the liver. It would be interesting to establish whether the same signals are operative in tissues in which both hypoxia and PPAR*γ* are present, like in adipose tissue in obesity [31, 32]. New studies are necessary to address the potential of new therapeutic tools able to counteract the pathophysiological processes leading to fibrosis.

## Figures and Tables

**Table 1:** Grades of fibrosis in rat liver.

Groups	*n*	Grades				
		0	1	2	3	4
Control	5	5 (100%)	0 (0%)	0 (0%)	0 (0%)	0 (0%)
CCl4 + FiO2 (0.21)	10	0 (0%)	2 (20%)	5 (50%)	2 (20%)	1 (10%)^a^
CCl4 + FiO2 (0.07)	10	0 (0%)	0 (0%)	4 (40%)	4 (40%)	2 (20%)^a,b^
CCl4 + FiO2 (0.07) + RSG (10 mg/kg)	10	0 (0%)	7 (70%)	3 (30%)	0 (0%)	0 (0%)^a,b,c^

^a^ *P* < 0.01 as compared with control group. ^b^*P* < 0.05 as compared with CCl4 + FiO2 (0.21) group. ^c^*P* < 0.05 as compared with CCl4 + FiO2 (0.07) group.

**Table 2:** Serum levels of HA, LN, PIIINP, and C IV (mean ± SD).

Groups	HA (*μ*g/L)	LN (*μ*g/L)	PIIINP (*μ*g/L)	C IV (*μ*g/L)
Control	85.45 ± 13.77	40.46 ± 9.74	21.02 ± 3.81	24.63 ± 6.72
CCl4 + FiO2 (0.21)	190.83 ± 20.23^a^	120.29 ± 22.59^a^	87.73 ± 15.82^a^	81.286 ± 15.28^a^
CCl4 + FiO2 (0.07)	256.29 ± 29.05^a,b^	188.08 ± 28.06^a,b^	142.16 ± 23.34^a,b^	119.36 ± 15.40^a,b^
CCl4 + FiO2 (0.07) + RSG (10 mg/kg)	182.73 ± 26.25^a,d^	103.63 ± 20.82^a,c,d^	72.39 ± 17.48^a,c,d^	62.27 ± 11.12^a,b,d^

^a^ *P* < 0.01 as compared with control group. ^b^*P* < 0.01 and ^c^*P* < 0.01 as compared with CCl4 + FiO2 (0.21) group. ^d^*P* < 0.01 as compared with CCl4 + FiO2 (0.07) group.
